# Annotations

**Published:** 1904-10-29

**Authors:** 


### Annotations.

### The Neglect of Medical Treatment.

In his recent presidential address delivered before the Clinical Society of London Dr. F. Taylor classified the contributions which have been made to the Society during the last 10 years and his analysis displays some rather interesting results. The papers on surgical subjects, it appears, slightly outnumbered those dealing with medical topics, but whilst three-fourths of the former dealt primarily with treatment, only one-fourth of the medical papers were mainly concerned with therapeutics. It will be admitted that this is a somewhat unfortunate state of affairs as it cannot be considered as other than indicative of a more or less general professional tendency. There are aspects of medical work which to many are much more attractive than the question of treatment, yet the latter necessarily remains as the main responsibility of the practitioner. It is not impossible that in some measure at least the neglect of treatment is due to the attitude adopted towards it in many of our medical schools where pathology and diagnosis are apt to be allowed to relegate it to an altogether subordinate position. Even in the portion of the curriculum specially devoted to it there is nowadays a disposition to cultivate a strictly scientific and often barren pharmacology at the expense of practical therapeutics. Yet when all these influences are discounted it is necessary to recognise the intrinsic difficulties of the subject more particularly in the direction of framing large and confident conclusions, and to admit that this consideration alone is sufficient to account for the dearth of contributions deplored by Dr. Taylor. It is, however, much to be desired that physicians shall be stimulated to consider the direct treatment of disease as distinct from the mere relief of symptoms. Possibly it is necessary to insist that much more precise and direct supervision of the details of treatment in individual cases is an essential condition of progress in this direction. The foundation of a special therapeutical society is an indication that this necessity is receiving some measure of attention and it will be a satisfactory result of Dr. Taylor's address if physicians generally are inclined to turn their minds more steadily towards measures and agents which are of value in the direct treatment of disease.

### The London School of Economics and Political Science.

The London School of Economics is, perhaps, less widely known than it deserves to be, in view of the fact that it has been in existence in the improved area at Clare Market for the last 10 years, and that its lectures give valuable training for the examinations of the University of London. Students at the school may be either internal or external, and as evening study counts for as much as that pursued during the day, the school offers advantages to those who, having completed their ordinary school studies, are engaged in bread-winning work. That there are many such who would be glad to train for the B.A., LL.B., or B.Sc. of London University is almost certain, and this school offers them an opportunity of completing their education. The subjects covered include sociology, economics, finance, law, and ethics, besides history and geography, and such helpful, if not definite, course of inquiry as is implied by the title "Suggested General Solutions of Social Problems." Of more definite pecuniary value, perhaps, is a course of instruction on library administration, and, in view of the number of free libraries which are being scattered broadcast all over the kingdom, there must be openings frequently arising for cultured young men and women to take charge of them. Those who have sought at the ordinary Public Library for help as to books on a given subject must know that librarians of such calibre are still scarce. Many of the lectures on political science would be of great value to factory and sanitary inspectors and to men and women desirous of obtaining appointments under Municipalities and the Local Government Board, while they would be interesting and helpful to all engaged in active philanthropic work. A course of lectures on factory legislation which is to be given by Miss B. L. Hutchins should have a special interest at the present moment, when all minds are turned to the problem of how the legislation of the last 60 years has affected the prosperity of the country and of our workers.

### A Hall Mark for Pills.

The newly constituted Institute of Hygiene is cultivating large ambitions in regard to the education and direction of public opinion in the various departments of hygiene. A scheme of lectures and examinations is to be developed, and is to include both London and the provinces. Upon these opinion may be reserved until the details are published. Another aspect of the Institute's activity is the foundation of a permament exhibition of appliances and substances claiming to be of value in the promotion of hygienic efficiency. To this no exhibit will be admitted until it has been examined by the governing body of the Institute, who must be satisfied that the proposed exhibit is really what it professes itself to be. The idea is that in existing circumstances both the medical profession and the public are so deluged with advertisements that they have neither time nor inclination to study the claims which these advance, and consequently no distinction is made between what is good and what is bad. Admission to the Institute's exhibition, we are told, will prevent this confusion by affording a competent and authoritative guarantee that materials so favoured have a definite value. This principle is to be applied in a catholic spirit, for the exhibition is to contain sanitary appliances, food products, beverages, medical specialities, clothing, and articles of domestic hygiene. Exhibits will receive from the Institute a certificate, which, however, remains in operation for only one year. The scheme is one which has obvious merits, and if worked in an impartial spirit ought to produce good results. There is no doubt that in this age of advertising the plain man is confused amidst the clamour of rival traders, and is thus apt to fall a victim to the one capable of exercising the most ingenious wiles. It may somewhat relieve this position to know that an organisation exists which professes to scrutinise the claims of the advertiser and to separate the sheep from the goats. The Institute's exhibition is situated in Devonshire Street, Portland Place, W.

